# Supplementary material for: Rapid Conversion of Amyloid-Beta 1-40 Oligomers to Mature Fibrils through a Self-Catalytic Bimolecular Process
Source: Int J Mol Sci. 2021 Jun 14;22(12):6370. doi: 10.3390/ijms22126370 (PMC8232289; doi:10.3390/ijms22126370)
Supplement: Supplementary file 1 [file ijms-22-06370-s001.zip › ijms-1234084-supplementary.pdf]

Supplementary information for:

Rapid conversion of amyloid-beta 1-40 oligomers to mature fibrils through a self-catalytic bimolecular process.

Bertrand Morel*, Maria P. Carrasco, Samuel Jurado & Francisco Conejero-Lara*

Material and Methods

Bis-ANS fluorescence spectroscopy

The fluorescence spectra of 10 μM 4,4′-Dianilino-1,1′-binaphthyl-5,5′-disulfonic acid (Bis-ANS) were recorded in the presence of Aβ oligomers either freshly purified or incubated for 1 day. Spectra were measured between 400 nm and 600 nm upon excitation at 350 nm using a Varian Cary Eclipse spectrofluorimeter (Agilent Technologies, Santa Clara, CA) equipped with a Peltier-controlled thermostatic cell holder.

Attenuated total reflectance Fourier-transform infrared (ATR-FTIR) spectroscopy

FTIR spectra were recorded at 37°C from 900 to 4000 cm^{-1} on a Bruker IFS-66 FTIR spectrometer (Bruker, Ettlingen, Germany) equipped with a liquid N2-cooled MCT detector and a BioATR-II cell. For each sample, 128 interferograms were co-added and Fourier transformed with a zero filling factor of 4 to yield spectra with a nominal resolution of 2 cm^{-1}. Buffer spectra were recorded under identical conditions and subtracted from the spectra of the protein sample. Spectral contributions from residual water vapor were reduced using the atmospheric compensation filter built in the Bruker OPUS software. In order to obtain secondary structure contents, the amide I band shape was fitted with a sum of Gaussian peaks using Origin 8.5 (OriginLab, Norhtampton, MA). The positions of the amide I band components were identified using the minima obtained from the second derivative of each spectra.

Circular dichroism spectroscopy

CD spectra were recorded in a Jasco J-715 spectropolarimeter (Jasco, Tokyo, Japan) equipped with Peltier-thermostatic cell holder. Measurements of the far-UV CD spectra (260–200 nm) were made with a 1 mm path length quartz cuvette at a protein concentration of ~80 μM in the case of LMW-Aβ40 and ~45 μM for p-Aβ40-OA. The resulting spectra were typically the average of 5 scans. Spectra were recorded at a scan rate of 100 nm/min, 1 nm step resolution, 1 s response, and 1 nm bandwidth. Each spectrum was corrected by baseline subtraction using the

blank spectrum obtained with the buffer and finally the CD signal was normalized to mean-residue molar ellipticity ([θ], in deg dmol^{-1} cm^{2}).

Mass spectrometry

The molecular weights of the F10-Aβ40-OA and F10-LMW-Aβ40 at 8 μM were performed by direct injection on a Synap G2 spectrometer (Waters) equipped with a quadrupole time of flight (Q-TOF) detector.

Intrinsic and di-tyrosine fluorescence

Tyrosine and dityrosine fluorescence spectroscopy experiments were performed using a Cary Eclipse Fluorescence Spectrophotometer (Varian Inc. Santa Clara, California, USA). For tyrosine fluorescence, the excitation wavelength was fixed to 280 nm and emission spectra were collected between 290 and 400 nm with slits width of 10 nm. Di-tyrosine fluorescence measurements were carried out using an excitation wavelength of 320 nm and spectra were collected from 340 nm to 500 nm using slits width of 10 nm.

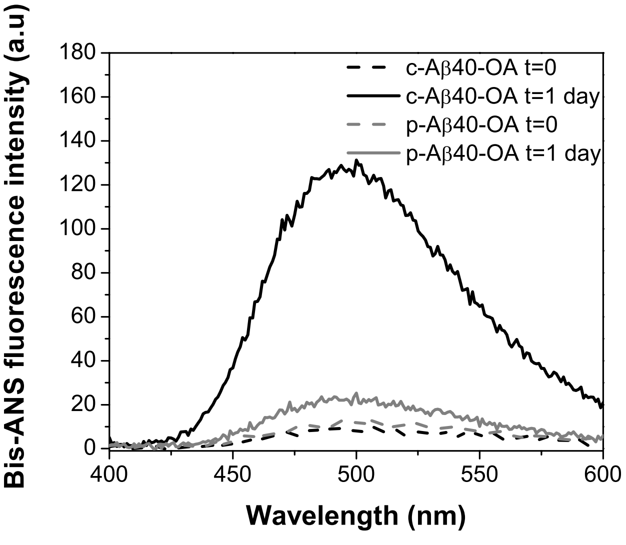

Figure S1a. Bis-ANS fluorescence scans of p-Aβ40-OA (grey) and c-Aβ40-OA oligomers (black) at 26 μM are represented before (dashed lines) and after incubation at 37°C for 1 day (continuous lines).

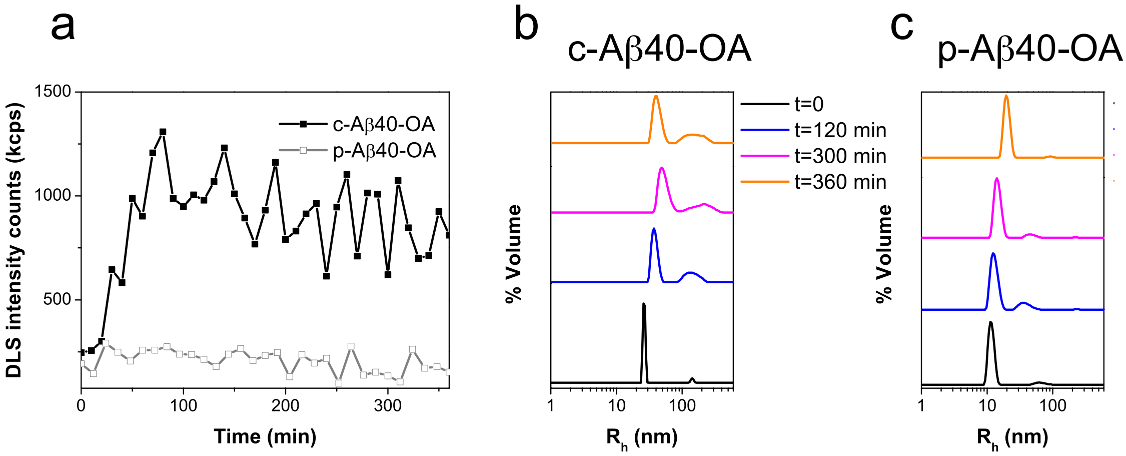

Figure S2. Aggregation time dependence of the different Aβ40 oligomer preparations followed by DLS. Variation of the scattering intensities as a function of the incubation time (a). Particle size distributions obtained by DLS for p-Aβ40-OA (c=25 μM) (b) and c-Ab40-OA (c=25μM) (c) freshly purified and during incubation at 37°C for different times.

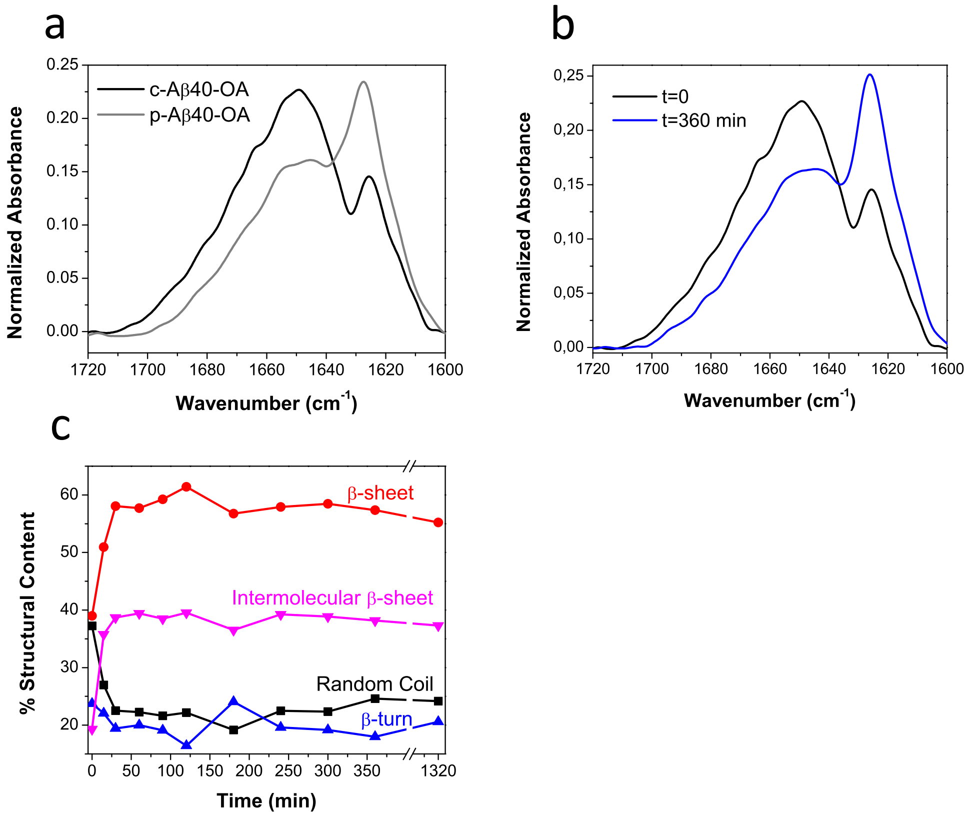

Figure S3. Changes of the secondary structure obtained by FTIR spectroscopy. Comparison of the FTIR spectra of p-Aβ40-OA (grey line) and c-Aβ40-OA (black line). Concentration of the samples was 25 μM (a). Incubation effect on the secondary structure of c-Aβ40-OA followed by FTIR (b). Changes in the content of secondary structure of c-Aβ40-OA during incubation at 37°C followed by ATR-FTIR (c).

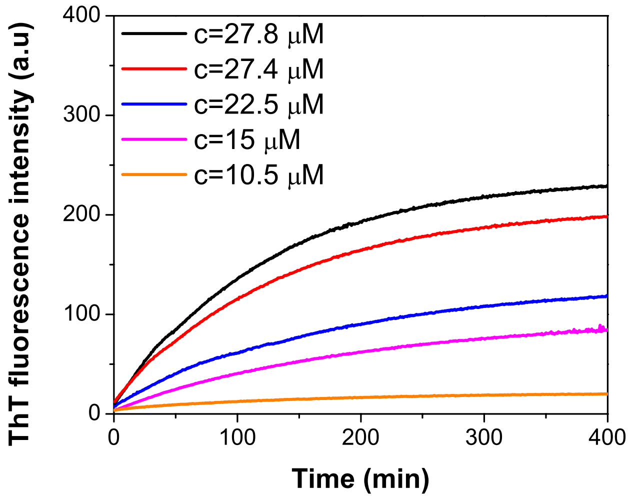

Figure S4. Concentration dependence of the aggregation kinetics of c-Aβ40-OA followed by ThT fluorescence at 37°C. Crude oligomers were prepared after incubation of LMW-Aβ40 for 4 hours. Oligomer concentrations are indicated in the graphs.

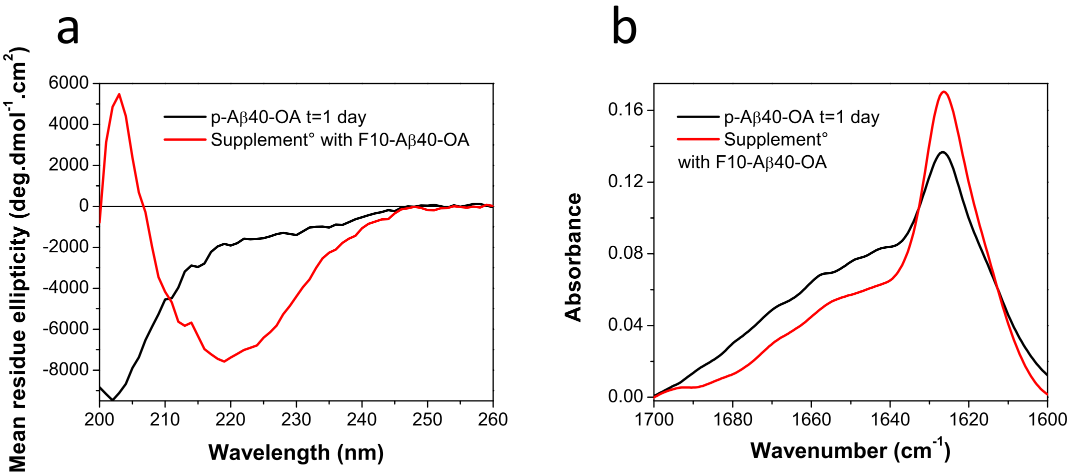

Figure S5. Effect of the supplementation with F10-Aβ40-OA on the secondary structure changes of p-Aβ40-OA at c=45 μM μM after overnight incubation followed by CD (a) and FTIR (b) spectroscopies. Samples were incubated at 37°C for about 360 minutes prior to supplementation with 1.9 μM of F10-Aβ40-OA.

a

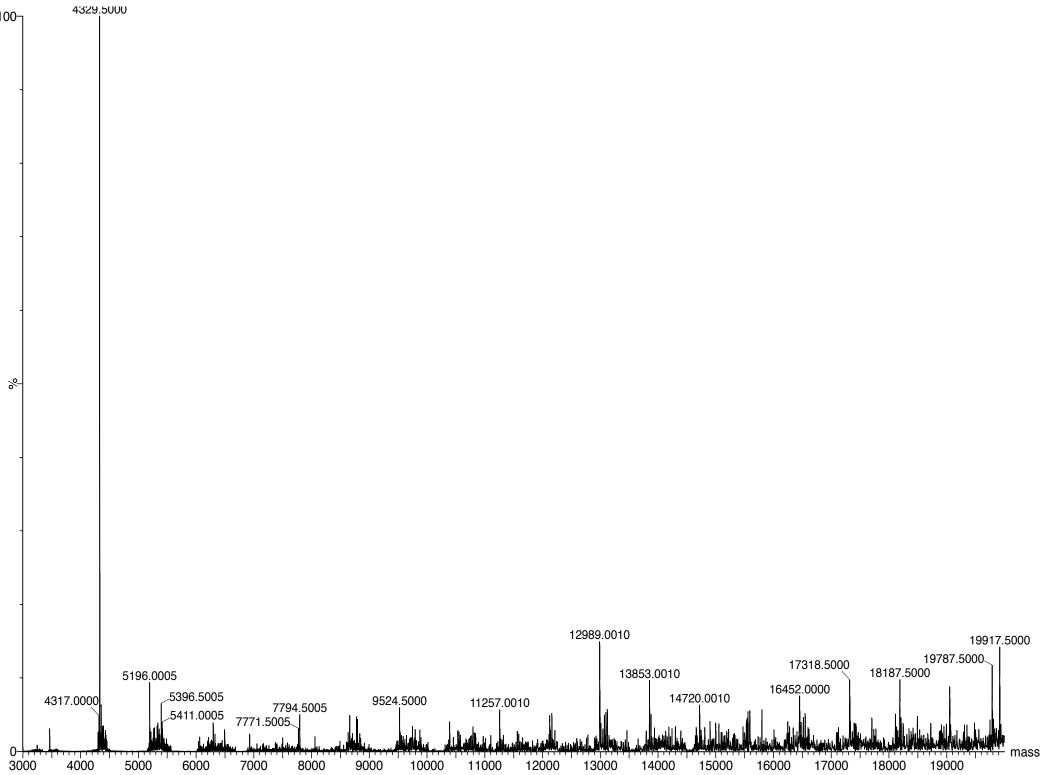
4329.5000
4317.0000
5196.0005
5396.5005
5411.0005
7771.5005
7794.5005
9524.5000
11257.0010
12989.0010
13853.0010
14720.0010
16452.0000
17318.5000
18187.5000
19787.5000
19917.5000
100
%
0
mass
3000 4000 5000 6000 7000 8000 9000 10000 11000 12000 13000 14000 15000 16000 17000 18000 19000

b

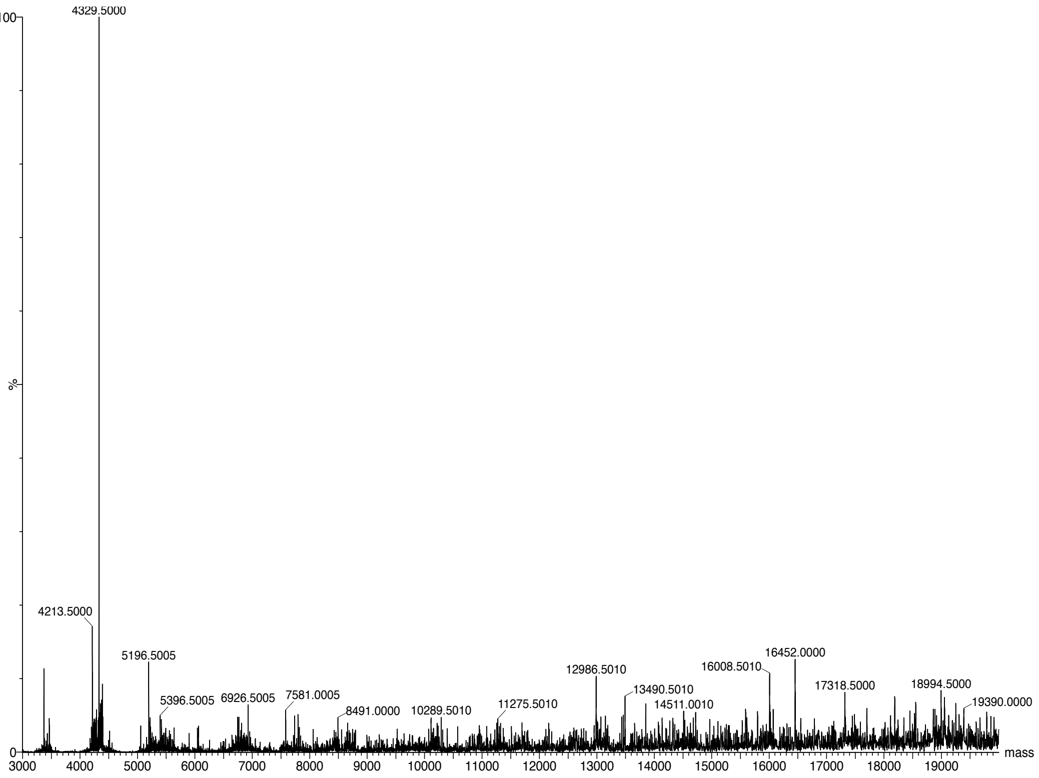
4329.5000
4213.5000
5196.5005
5396.5005
6926.5005
7581.0005
8491.0000
10289.5010
11275.5010
12986.5010
13490.5010
14511.0010
16008.5010
16452.0000
17318.5000
18994.5000
19390.0000
100
%
0
mass
3000 4000 5000 6000 7000 8000 9000 10000 11000 12000 13000 14000 15000 16000 17000 18000 19000

Figure S6. Mass spectra obtained from direct injection on Q-TOF ESI of F10-LMW-Aβ40 (a) and F10-Aβ40-OA (b) at 8 μM.

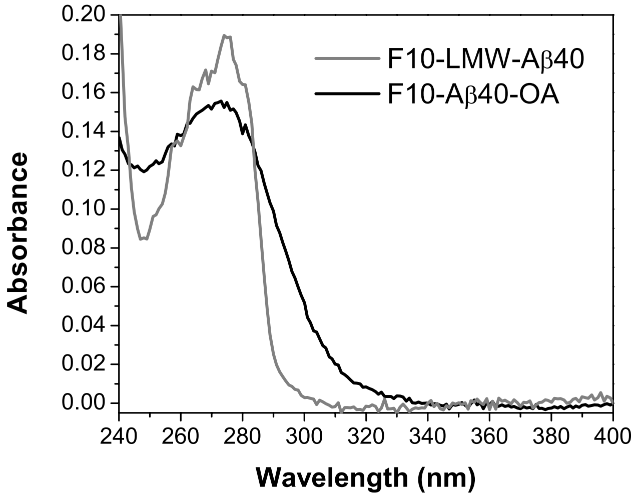

Figure S7. UV-vis spectra of F10-LMW-Aβ40 (grey line) and F10-Aβ40-OA (black line).

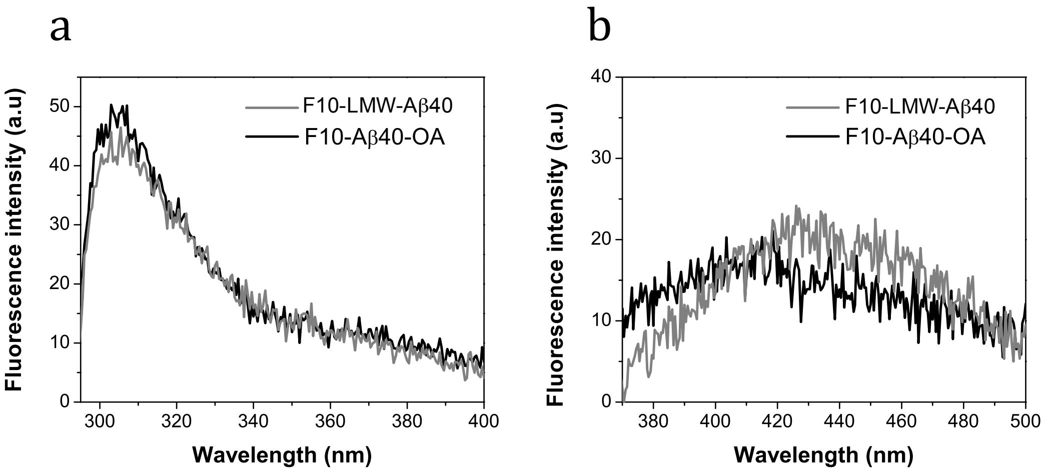

Figure S8. Intrinsic tyrosine (a) and dityrosine (b) fluorescence spectra of F10-LMW-Aβ40 (grey lines) and F10-Aβ40-OA (black lines) at 9.4 μM.

Table S1. Secondary structure analysis of the oligomers derived from the deconvolution of the amide I regions of the FTIR spectra at t0 and after 400 min of incubation at 37°C.

	c-Aβ40-OA		p-Aβ40-OA*	
	0	400 min	0	400 min
Random Coil (%)[a]	37.3	26.2	15.9	-
β-sheets (%)[b]	39	59.2	44.0	72.9
β-turns (%)[c]	23.7	14.6	40.1	27.1
Intermolecular β-sheets (%)[d]	19.3	38.0	37.3	46.7

* data taken from [1].

** Characteristics Amide I wavenumbers obtained from [2]

[a]: Random coil : 1645 cm^{-1} – 1655 cm^{-1}

[b]: β-sheet : 1620 cm^{-1} - 1640 cm^{-1} ; 1670 cm^{-1} - 1695 cm^{-1}

[c]: β-turns : 1685 cm^{-1} - 1655 cm^{-1}

[d]: Intermolecular β-sheet : 1627 cm^{-1} - 1615 cm^{-1}

References

1. B. Morel, M. P. Carrasco, S. Jurado, C. Marco and F. Conejero-Lara, *Phys. Chem. Chem. Phys.*, 2018, **20**, 20597-20614.
2. S. A. Tatulian, *Methods Mol. Biol.*, 2013, **974**, 177-218.
